# Chloroplast genomic diversity in eleven Pennisetum species

**DOI:** 10.3389/fgene.2025.1594209

**Published:** 2026-01-12

**Authors:** Muhan He, Qi Wang, Yuwen Zhang, Chunjuan Shi

**Affiliations:** Yunnan Forestry Technological College, Kunming, Yunnan, China

**Keywords:** Pennisetum, chloroplast genome, codon bias, genetic diversity, SSR

## Abstract

**Background:**

This study presents a comprehensive analysis of the chloroplast genomes from eleven Pennisetum species, including major and minor millets. Through sequencing and assembly, we characterized the chloroplast genomes, revealing conserved gene content and structure while also identifying species-specific variations and mutational biases that suggest adaptive evolutionary processes.

**Results:**

Our findings demonstrate a general preference for A/T-ending codons in line with the AT-rich nature of chloroplast DNA, as evidenced by Relative Synonymous Codon Usage analysis, which indicates codon usage bias and hints at evolutionary pressures for translational efficiency. Intron analysis within these genomes unveiled considerable variability, reflecting the genomes’ evolutionary flexibility and potential for adaptation to diverse environmental conditions. Comparative genomic analyses elucidated the phylogenetic relationships among the Pennisetum species, aligning with known taxonomic classifications and uncovering new evolutionary insights. Additionally, the study identified numerous simple sequence repeats, providing valuable markers for further genetic and phylogenetic research.

**Conclusion:**

Overall, this research enhances our understanding of chloroplast genome evolution in Pennisetum, providing a valuable genetic resource that contributes to botanical and agricultural knowledge while underscoring the broader significance of plastome studies in elucidating plant evolutionary mechanisms.

## Background

The genus Pennisetum, a member of the tribe Paniceae within the family Poaceae, comprises approximately 140 species distributed across tropical and subtropical regions globally (Brunken, 1977). This genus, segmented into three sections—Sect. Gymnothrix, Sect. Penicillaria, and Sect. Pennisetum—encompasses species that serve as vital grain and forage crops in Africa and Asia (Abdi et al., 2019). Pennisetum species such as *P. glaucum*, *P. purpureum*, *P. ramosum*, *P. orientale*, and *P. clandestinum* are notable for their economic importance in Africa and Asia, fulfilling roles ranging from food sources to ornamental and pasture plants (Abdi et al., 2019). These species exhibit robust adaptability to diverse environmental conditions (Kumar et al., 2019; Yadav et al., 2012; Shivhare and Lata, 2017; Sharma et al., 2021), including low-fertility soils, and present a variety of reproductive behaviors, chromosome numbers, ploidy levels, and life cycles (Yadav et al., 2024; Martel et al., 1997; Burton et al., 1968).

The chloroplast genome has been recognized for its conserved gene content and organization, offering a stable marker for phylogenetic studies (Daniell et al., 2016). The chloroplast, pivotal in converting solar energy into chemical energy via photosynthesis, possesses its distinct DNA, typically spanning 120–180 kilobases in higher plants (Li D.-M. et al., 2020). Characterized by its minimalistic architecture and presence in multiple copies, the chloroplast genome is delineated by a circular quadripartite structure comprising a large single-copy (LSC) region, a small single-copy (SSC) region, and two inverted repeat (IR) sections (Meng et al., 2018; Mader et al., 2018). The inception of chloroplast genome sequencing was marked by liverwort and tobacco studies in 1986 (Shinozaki et al., 1986; Ohyama et al., 1986). Advances in sequencing technologies, particularly have since streamlined and reduced the cost of chloroplast genome sequencing (Godden et al., 2012). These genomes, rich in functional genes, are increasingly recognized for their significance in species identification and evolutionary biology studies (Daniell et al., 2016; Godden et al., 2012; Presting, 2006; Zurawski and Clegg, 1987; De Las Rivas et al., 2002). The analytical exploration of these genomes has enhanced our understanding of plant phylogeny and the intricacies of plant evolutionary mechanisms (Tonti Filippini et al., 2017; Howe et al., 2003).

The analysis of chloroplast genomic diversity across related species offers invaluable insights into their evolutionary trajectories, genetic divergence, and adaptive mechanisms. Simple sequence repeats (SSRs), or microsatellites, are tandemly repeated stretches of short nucleotide motifs widely dispersed throughout genomes, including the chloroplast (Gupta et al., 2021; Wang et al., 2020; Li C. et al., 2020). These repetitive sequences are highly polymorphic and considered informative molecular markers, rendering them advantageous for assessing genetic diversity, population genetics, and phylogenetic relationships (Haq et al., 2021; Sathyamurthy et al., 2024). Moreover, the characterization of SSRs in chloroplast genomes can shed light on the dynamics of genome evolution and provide a means for species delimitation and identification (Dong et al., 2012; Tang et al., 2022; Li et al., 2015).

Through the comparative genomic analysis, we aimed to investigate the Chloroplast genomes of *P. longissimum*, *P. purpureum* Schumach, *P. massaicum* Stapf, *P. clandestinum*, *Eragrostis nutans*, *P. villosum*, *P. setaceum*, *P. orientale*, *P. polystachion*, and *P. glaucum* ‘Purple Majesty’, providing significant insights into the chloroplast genome evolution in these species and differences. The findings are expected to contribute significantly to the existing knowledge of chloroplast genome evolution in Pennisetum and provide a valuable genetic resource for further studies on this important genus.

## Methods

### Plant materials and DNA extraction

Seeds of *Pennisetum longissimum*, *P. purpureum* Schumach, *P. massaicum* Stapf, *P. clandestinum*, *Eragrostis nutans*, *P. villosum*, *P. setaceum*, *P. orientale*, *P. polystachion*, and *P. glaucum* ‘Purple Majesty’ were obtained from the Houshan Base of Yunnan Forestry Vocational and Technical College in April 2022 (voucher numbers: Yfvtc10457765, Yfvtc10457766, Yfvtc10457767, Yfvtc10457768, Yfvtc10457769, Yfvtc10457770, Yfvtc10457771, Yfvtc10457772, Yfvtc10457773, Yfvtc10457774, respectively).

The seeds were germinated and grown in controlled laboratory conditions until the plants developed sufficient leaf material for DNA extraction. Fresh leaves from each plant were used to extract total genomic DNA utilizing the protocol described by Li et al., (Xu et al., 2021). The quality and quantity of extracted DNA were assessed using 1.0% agarose gel electrophoresis with SYBR Green I staining.

### Illumina sequencing and assembly

DNA libraries with 350-bp insert sizes were prepared using the Nextera XT DNA Library Prep Kit (Illumina Inc., San Diego, CA, United States), following the manufacturer’s guidelines. Sequencing was conducted on the Illumina HiSeq X-ten platform to generate 150-bp paired-end reads. Raw sequencing data were processed and trimmed using Trimmomatic v0.32 (Bolger et al., 2014) to obtain clean reads, which were then assembled into contigs using SPAdes 3.6.1 (Bankevich et al., 2012) with a range of K-mer parameters. BLAST searches were employed to identify contigs related to chloroplast genomes, which were further assembled into complete genomes using Sequencher 5.4.5 (Gene Codes, Ann Arbor, MI).

### Annotation and comparative analysis

The chloroplast genomes were annotated with Plann (Huang and Cronk, 2015), using the *P. glaucum* chloroplast genome as a reference. Structural features were visualized with OGDRAW software (Greiner et al., 2019). Codon usage bias was analyzed with CodonW software (Sharp and Li, 1987), particularly focusing on Relative Synonymous Codon Usage (RSCU). Comparative genomic analysis across the 11 Pennisetum species was executed using the mVISTA program (Frazer et al., 2004).

### Analysis of simple sequence repeats

Chloroplast SSRs were identified using GMATA software (Wang and Wang, 2016), setting repeat number thresholds for various SSR types. The chloroplast genomes were scanned for forward, reverse, palindromic, complementary, and tandem repeats using REPuter (Kurtz et al., 2001) with specified parameters. Tandem Repeats Finder was employed to pinpoint tandem repeats, and the distribution of SSRs in different genomic regions was thoroughly examined.

## Results

### Overview of chloroplast genomes

This study presents a comprehensive overview of the chloroplast genomes across eleven Pennisetum species, revealing insights into their genomic characteristics. The species analyzed include *P. longissimum, P. purpureum Schumach, P. massaicum Stapf, P. clandestinum, Eragrostis nutans, P. villosum, P. setaceum, P. orientale, P. polystachion, P. glaucum ‘Purple Majesty’*, each showcasing unique genomic features.

For *P. longissimum*, a total of 50,666,080 reads were generated, with 50,655,502 clean reads leading to 7,527,043,920 clean bases (Table 1; Supplementary Figure S1). The GC content was noted to be 45.39%, with a high-quality score percentage (>Q20) of 94.45% and >Q30 of 82.84%. In contrast, *P. purpureum* Schumach produced 47,109,166 total reads, with 47,098,422 clean reads and 7,024,869,804 clean bases, showing slightly higher GC content at 45.43%.

**TABLE 1:** Overview of sequencing output and quality metrics for Pennisetum species.

Samples	Total reads	Clean reads	Clean bases	GC content	%>Q20	%>Q30
*P. longissimum*	50,666,080	50,655,502	7,527,043,920	45.39%	94.45%	82.84%
*P. purpureum*	47,109,166	47,098,422	7,024,869,804	45.43%	94.52%	82.95%
*P. massaicum*	48,009,716	47,999,486	7,078,691,044	45.56%	94.31%	82.37%
*P. clandestinum*	51,070,350	51,059,170	7,598,650,180	45.05%	94.39%	82.62%
*Eragrostis nutans*	54,485,434	54,473,774	8,115,540,002	44.84%	94.81%	83.82%
*P. villosum*	54,633,212	54,622,304	8,078,326,328	44.96%	94.19%	81.97%
*P. setaceum*	50,324,114	50,313,252	7,388,940,252	44.78%	94.61%	83.22%
*P. orientale*	46,762,034	46,752,548	6,941,460,868	45.95%	93.89%	81.27%
*P. polystachion*	26,262,578	26,213,588	3,915,408,662	48.15%	96.02%	87.19%
*P. clandestinum*	23,855,058	23,817,774	3,545,349,846	45.98%	95.59%	86.13%
*P. glaucum*	22,959,652	22,915,742	3,422,978,418	47.80%	96.12%	87.48%

*P. massaicum* Stapf generated 48,009,716 reads, with 47,999,486 clean reads and a total of 7,078,691,044 clean bases (Table 1). This species exhibited a GC content of 45.56%, slightly higher than the previous species, and quality scores of 94.31% (>Q20) and 82.37% (>Q30). *P. clandestinum* had 51,070,350 total reads, with 51,059,170 clean reads and 7,598,650,180 clean bases, with a GC content of 45.05%.

*E. nutans* showed the highest number of total reads among the analyzed species at 54,485,434, with 54,473,774 clean reads and 8,115,540,002 clean bases. It also had the lowest GC content at 44.84% but the highest quality scores with >Q20 at 94.81% and >Q30 at 83.82%.

The sequencing efforts resulted in a high volume of total reads across all species, demonstrating the efficacy of the sequencing technology. The conversion from total to clean reads shows excellent data quality, with very high percentages of clean reads (>99% in all cases). The GC content varied across the species, ranging from 44.84% to 48.15%, which is within the expected range for plant chloroplast genomes, indicating good representation of genomic content. Quality metrics, including > Q20 and >Q30 scores, were notably high across all species, with >Q30 values mostly exceeding 80%. These high-quality scores signify that the majority of the sequencing output is highly reliable and accurate, minimizing the possibility of errors in downstream analyses. The species *P. polystachion*, *P. clandestinum* Hoohst, and *P. glaucum* ‘Purple Majesty’ displayed particularly high GC content and quality scores, indicating exceptionally good sequencing outcomes. The consistent high-quality data across the species provides a robust foundation for comprehensive genomic and evolutionary studies in the Pennisetum genus.

The chloroplast genome sizes range from 134,829 bp in *P. glaucum* ‘Purple Majesty’ to 138,571 bp in *P. orientale*, with the majority of the genomes being around 138,000 bp in length (Table 2). The overall GC content is relatively consistent, ranging from 38.2% in *P. glaucum* ‘Purple Majesty’ to 38.67% in *P. clandestinum* and *P. longissimum*, with slightly higher GC content in the inverted repeat (IR) regions. The chloroplast genomes are typically divided into four regions: the large single-copy (LSC) region, the small single-copy (SSC) region, and two inverted repeat (IR) regions. The percentage contribution of each region to the overall genome size is relatively consistent across the genomes, with the LSC region contributing around 54–55%, the SSC region contributing around 41–42%, and the IR region contributing around 1.6–1.7% for most species. However, *P. glaucum* ‘Purple Majesty’ is an exception, with the LSC region contributing 58.67%, the SSC region contributing 8.98%, and the IR region contributing 16.17% of the total genome size. The lengths of these regions also vary, with the LSC region ranging from 73,712 bp to 81,033 bp, the SSC region ranging from 12,406 bp to 57,697 bp, and the IR region ranging from 2,297 bp to 22,374 bp across the analyzed genomes.

**TABLE 2:** Comparative genomic features of Pennisetum chloroplast genomes.

Samples	*P. longissimum*			*P. purpureum* schumach			*P. massaicum* stapf		
Category	Length (bp)	GC (%)	Percentage (%)	Length (bp)	GC (%)	Percentage (%)	Length (bp)	GC (%)	Percentage (%)
Genome	138,134	38.62	100	138,173	38.6	100	137,931	38.61	100
LSC	76,036	39.75	55.05	76,146	39.72	55.11	75,596	39.81	54.81
SSC	57,368	36.89	41.53	57,299	36.89	41.47	57,593	36.79	41.76
IR	2,365	41.55	1.71	2,364	41.4	1.71	2,371	41.44	1.72

Samples	*P. clandestinum*			*Eragrostis nutans*			*P. villosum*		
Category	Length (bp)	GC (%)	Percentage (%)	Length (bp)	GC (%)	Percentage (%)	Length (bp)	GC (%)	Percentage (%)
Genome	138,003	38.66	100	134,829	38.2	100	138,571	38.6	100
LSC	75,950	39.8	55.04	73,712	39.31	54.67	57,843	36.84	41.74
SSC	57,323	36.92	41.54	56,417	36.48	41.84	76,134	39.75	54.94
IR	2,365	41.5	1.71	2,350	41.51	1.74	2,297	41.69	1.66

Samples	*P. setaceum*			*P. orientale*			*P. polystachion*		
Category	Length (bp)	GC (%)	Percentage (%)	Length (bp)	GC (%)	Percentage (%)	Length (bp)	GC (%)	Percentage (%)
Genome	138,037	38.67	100	138,408	38.63	100	138,171	38.59	100
LSC	76,082	39.79	55.12	75,981	39.78	54.9	76,132	39.71	55.1
SSC	57,227	36.95	41.46	57,697	36.89	41.69	57,311	36.87	41.48
IR	2,364	41.44	1.71	2,365	41.42	1.71	2,364	41.31	1.71

Samples	*P. clandestinum* Hoohst			*P. glaucum* ‘purple majesty’		
Category	Length (bp)	GC (%)	Percentage (%)	Length (bp)	GC (%)	Percentage (%)
Genome	138,003	38.66	100	138,119	38.59	100
LSC	80,849	36.54	58.59	81,033	36.47	58.67
SSC	12,406	32.97	8.99	12,408	32.99	8.98
IR	22,374	44.07	16.21	22,339	43.99	16.17

*Distribution of LSC, large single-copy; SSC, small single-copy; and IR, inverted repeat; regions across the studied species.

### Gene prediction across Pennisetum chloroplast genomes

The comparative analysis of gene prediction in the 11 chloroplast genomes of Pennisetum species reveals a high degree of conservation across the majority of genes (Table 3; Supplementary Figures S1–S11; Supplementary Table S1). All species possess a highly conserved set of genes involved in photosynthesis, including subunits of ATP synthase, photosystem II, NADH-dehydrogenase, cytochrome b/f complex, photosystem I, and the large subunit of RuBisCO (rbcL). The genes involved in self-replication, such as ribosomal RNAs, DNA-dependent RNA polymerases, and ribosomal proteins, are also highly conserved. Several other genes encoding proteins like c-type cytochrome synthesis, envelope membrane protein, protease, translational initiation factor, and maturase are present in most or all species. However, there are a few unique genes specific to certain species. For instance, *P. purpureum* is the only species lacking the psbN gene, which encodes a subunit of photosystem II. Additionally, *Eragrostis nutans* and *P. setaceum* contain an extra copy of the rpoC2 gene, encoding a subunit of RNA polymerase. The chloroplast genomes also contain conserved open reading frames (ORFs) like ycf4, ycf2, and ycf15, whose functions are not well characterized, with some variation in their presence among species.

**TABLE 3:** Gene content and organization in Pennisetum chloroplast genomes.

Samples	*P. longissimum*			*P. purpureum* schumach			*P. massaicum*		
Category	Length (bp)	GC (%)	Percentage (%)	Length (bp)	GC (%)	Percentage (%)	Length (bp)	GC (%)	Percentage (%)
CDS	57,617	39.12	41.71	56,305	39.13	40.75	58,383	39.06	42.33
rRNA	8,804	54.75	6.37	9,200	54.67	6.66	9,198	54.7	6.67
tRNA	2,946	52.72	2.13	2,785	52.96	2.02	2,968	52.66	2.15
Intergenic	53,079	34.67	38.43	54,064	34.56	39.13	52,095	34.47	37.77
Intron	15,688	38.49	11.36	15,819	38.65	11.45	15,287	38.58	11.08

Samples	*P. clandestinum*			*Eragrostis nutans*			*P. villosum*		
Category	Length (bp)	GC (%)	Percentage (%)	Length (bp)	GC (%)	Percentage (%)	Length (bp)	GC (%)	Percentage (%)
CDS	59,297	39.17	42.97	60,637	38.8	44.97	58,710	39.12	42.37
rRNA	9,200	54.67	6.67	9,208	54.75	6.83	9,198	54.65	6.64
tRNA	3,030	52.48	2.2	2,905	52.87	2.15	2,968	52.76	2.14
Intergenic	49,791	34.23	36.08	45,731	33.14	33.92	52,346	34.38	37.78
Intron	16,685	38.73	12.09	16,348	38.24	12.12	15,349	38.63	11.08

Samples	*P. setaceum*			*P. orientale*			*P. polystachion*		
Category	Length (bp)	GC (%)	Percentage (%)	Length (bp)	GC (%)	Percentage (%)	Length (bp)	GC (%)	Percentage (%)
CDS	57,383	39.21	41.57	61,020	39.19	44.09	59,210	39.1	42.85
rRNA	8,804	54.75	6.38	9,196	54.67	6.64	9,198	54.65	6.66
tRNA	2,946	52.72	2.13	2,903	52.91	2.1	2,964	52.73	2.15
Intergenic	52,961	34.65	38.37	48,615	34.02	35.12	50,184	34.19	36.32
Intron	15,943	38.62	11.55	16,674	38.72	12.05	16,615	38.62	12.02

Samples	*P. clandestinum*			*P. glaucum*		
Category	Length (bp)	GC (%)	Percentage (%)	Length (bp)	GC (%)	Percentage (%)
CDS	59,333	39.16	42.99	59,246	39.09	42.89
rRNA	9,200	54.67	6.67	9,198	54.65	6.66
tRNA	3,030	52.44	2.2	2,964	52.7	2.15
s	49,755	34.23	36.05	50,096	34.2	36.27
Intron	16,685	38.75	12.09	16,615	38.62	12.03

### Intron characteristics in Pennisetum chloroplast genomes

The comprehensive analysis of intron positions and lengths within the chloroplast genomes of 11 Pennisetum species revealed significant variability across various genes. The study encompassed genes such as trnA-UGC, trnI-GAU/CAU, trnK-UUU, rps16, trnG-UCC, atpF, ycf3, trnL-UAA, trnV-UAC, rpl16, petD, petB, rpl2, and rpoC2, among others.

In general, most genes contained one or more introns, with the position and size of these introns showing considerable diversity among the genes and species. The trnK-UUU gene was notable for its large intron size, which varied from 2,455 bp to 2,513 bp across the genomes. This size variation underscores the dynamic nature of intron evolution in chloroplast genomes. Introns in the chloroplast genomes exhibited a wide range of lengths. For instance, some species displayed relatively short introns like the 33 bp intron in the rpoC2 gene, while others had much longer introns, such as the 2,467 bp intron in the trnK-UUU gene of *E. nutans*. This diversity in intron length is indicative of the complex evolutionary pressures and functional dynamics at play within the chloroplast genomes. The results also highlighted species-specific intronic features, pointing to unique evolutionary trajectories. For example, additional introns in certain genes and the complete absence of specific genes in some species reflect the intricate patterns of genome evolution and adaptation in the chloroplasts of these plants.

The dataset from Supplementary Table S2 provides a valuable foundation for understanding the intricacies of intron dynamics and their evolutionary and functional implications in the chloroplast genomes of Pennisetum species. These findings contribute to our broader knowledge of genomic organization and evolution in plant chloroplasts, offering potential insights into gene regulation and expression mechanisms within these organelles.

### Comparative analysis of codon usage Pennisetum chloroplast genomes

The codon usage patterns across the chloroplast genomes of 11 Pennisetum species, as detailed in Supplementary Table S3, demonstrate a balance of genomic conservation and variability. These patterns reveal a strategic preference for certain codons which is likely a result of evolutionary pressures to optimize the chloroplast’s translational efficiency, crucial for its photosynthetic function. The RSCU analysis across the chloroplast genomes of the 11 Pennisetum species, uncovers codon usage biases essential for understanding the translational intricacies of these plants. The RSCU values, which normalize the frequency of synonymous codons, highlight a pronounced preference for codons ending in adenine (A) or thymine (T), corroborating the AT-rich genomic environment of chloroplast DNA. This preference is not uniform across all species, indicating a delicate balance between genomic conservation and species-specific evolutionary adaptations.

Significant findings from the RSCU data show that while some codons such as AAA (encoding lysine) consistently show high RSCU values, indicating a strong bias towards this codon in the chloroplast genomes of these species. Conversely, codons like CGC (encoding arginine) are less favored, displaying lower RSCU values, which suggests a selective constraint or functional adaptation affecting these codon choices.

The analysis unveils that while there is a general trend towards certain codon preferences, species-specific variations exist, underscoring the evolutionary diversity within the Pennisetum genus. These variations could be linked to the species’ distinct ecological niches and evolutionary trajectories, highlighting the role of natural selection in shaping chloroplast genome evolution. For example, genes critical for photosynthesis and stress responses might exhibit unique codon preferences to ensure rapid and accurate protein synthesis under variable environmental stresses.

### SSR analysis

The analysis of simple sequence repeat (SSR) markers identified in the chloroplast genomes of 11 plant species revealed several notable findings (Supplementary Table S4). Across the analyzed genomes, the most abundant SSR motifs were mononucleotide repeats (A/T) and dinucleotide repeats (AT/TA), while trinucleotide and tetranucleotide repeats were less common, with only a few instances observed in some species.

In terms of SSR marker frequency, *Pennisetum longissimum*, *P. purpureum*, and *P. massaicum* exhibited the highest numbers, with a total of 30, 33, and 35 markers, respectively. *P*. *clandestinum*, *E*. *nutans*, and *P. villosum* had moderate numbers of SSR markers, ranging from 41 to 29. *P. setaceum*, *P. orientale*, *P. polystachion*, and *P. glaucum* exhibited relatively lower numbers of SSR markers, ranging from 29 to 35. The majority of the identified SSRs had repeat lengths ranging from 10 to 16 nucleotides, while longer repeats (≥20 nucleotides) were rare and observed only in a few species, such as *P. setaceum* and *P. orientale*.

Comparative analysis of the SSR profiles revealed interesting insights. The number and distribution of SSR markers varied among the analyzed species, suggesting potential intraspecific variation in the chloroplast genomes. Species belonging to the same genus exhibited both similarities and differences in their SSR profiles, indicating potential evolutionary divergence. The observed differences in SSR marker abundance and composition among the species suggest interspecific variation in the chloroplast genomes, which could be attributed to factors such as evolutionary history, genome size, and adaptations to different environmental conditions.

These identified SSR markers can serve as valuable molecular markers for population genetics studies, species identification, and phylogenetic analyses within these plant groups. The observed variations in SSR profiles can be further explored to understand the genetic diversity and evolutionary relationships among these species. Additionally, further validation and characterization of the identified SSR markers may be necessary for their effective utilization in various applications.

## Discussion

The detailed comparative analysis of chloroplast genomes across 11 Pennisetum species has unveiled patterns of genetic conservation and divergence, shedding light on the complex evolutionary processes that shape these essential genomic elements. The chloroplast genomes exhibit a conserved structure, typical of the Poaceae family (Doyle et al., 1992; Cahoon et al., 2010; Huang et al., 2017), but also reveal species-specific variations that likely represent adaptations to diverse ecological niches and environmental pressures (Hu et al., 2022; Hu et al., 2015).

The successful sequencing and assembly of eleven Pennisetum chloroplast genomes, validated by the high-quality metrics presented in Table 1, provided a solid foundation for this comparative study. The structural features of these genomes, detailed in Table 2, largely conform to the conserved organization observed across the Poaceae family, with a typical quadripartite structure and consistent GC content (Doyle et al., 1992; Cahoon et al., 2010; Huang et al., 2017). This underlying conservation reflects the essential and constrained functions of the chloroplast. However, our analysis also revealed significant species-specific variations, such as the substantial IR region expansion in *P. clandestinum* Hoohst and *P. glaucum* “Purple Majesty”. Such structural rearrangements, along with variations in gene content and intron lengths, highlight the dynamic evolutionary processes that shape organellar genomes, even within a closely related group of species. These variations not only serve as valuable phylogenetic markers but also suggest potential adaptations to diverse ecological niches (Hu et al., 2022; Hu et al., 2015).

Our comparative analysis allows us to draw direct links between genomic features and the evolutionary history of these species. The phylogenetic relationships inferred from the complete chloroplast genomes are strongly corroborated by specific patterns of variation in gene content, intron structure, and repetitive elements. For example, our SSR analysis revealed that *P. longissimum*, *P. purpureum*, and *P. massaicum* exhibited the highest numbers of SSR markers (30, 33, and 35, respectively). This shared characteristic of high SSR density suggests a closer evolutionary relationship among these species, a hypothesis supported by their clustering in phylogenetic analyses of the Poaceae family. Conversely, the more moderate SSR counts in species like *P. clandestinum* and *P. villosum* reflect their distinct evolutionary trajectories.

Furthermore, specific gene content variations serve as clear markers of evolutionary divergence. The lack of the psbN gene, which encodes a photosystem II subunit, is a unique feature observed only in *P. purpureum* among the studied species. This gene loss likely represents a key evolutionary event (a putative synapomorphy) that occurred after its divergence from a common ancestor. Similarly, the presence of an extra copy of the rpoC2 gene in *E. nutans* and *P. setaceum* distinguishes them from the other analyzed species and provides a valuable marker for phylogenetic delimitation.

Intron characteristics also provide evidence for species divergence. The trnK-UUU gene, for instance, contains a large intron that varies in size from 2,455 bp to 2,513 bp across the genomes. While this variation underscores the dynamic nature of intron evolution, the degree of size difference is smaller among more closely related species, suggesting that intron length evolution correlates with species divergence rates.

The size and gene content of the chloroplast genomes were found to be relatively consistent, reflecting the stable inheritance and functional constraints of these organelles. However, the presence of unique genes and variations in intron lengths and configurations across different species suggest a dynamic evolutionary history. For instance, the large intron in the trnK-UUU gene and the variable intron sizes in the rpoC2 gene highlight the potential for structural modifications that could impact gene expression regulation and chloroplast function (Zurawski and Clegg, 1987; Xiao-Ming et al., 2017; Borsch and Quandt, 2009).

In seed-bearing plants, the bias in nucleotide mutations plays a crucial role in shaping codon variability, significantly affecting the genetic makeup (Zhou et al., 2008). Research on the chloroplast genome within the Poaceae family has underscored the profound effect that mutational bias exerts on codon preferences and overall genetic evolution (Wang et al., 2022; Zhang et al., 2016; Zhang et al., 2012). The preference for certain amino acids in these organisms often mirrors the selective pressures on protein functionalities (McInerney, 1998). To quantify codon bias while discounting the amino acid composition’s effects, Relative Synonymous Codon Usage (RSCU) analysis is employed. For instance, in *P. glaucum*, studies have identified that 29 codons exhibit RSCU values greater than 1, denoting a distinct bias in their usage, which is critical for understanding the molecular evolution and adaptive strategies of these plants (Raveendar et al., 2019). The analysis of RSCU revealed a clear preference for A/T-ending codons, consistent with the overall AT richness of the chloroplast DNA. However, Sathyamurthy et al. (2024) suggested codon bias for isoleucine (I), the codon ATT was found to have a prominent codon usage bias, indicating a strong preference for this codon in encoding isoleucine. In contrast, the codon TGC, which encodes cysteine (C), showed the least preference. This codon usage bias may be an evolutionary response to optimize the translational efficiency of essential photosynthetic proteins. Intriguingly, the RSCU analysis also pointed out specific codons that deviate from this trend, suggesting possible areas of selection pressure or gene-specific adaptations.

The functional implications of the observed genomic variations are profound. They not only reflect the evolutionary history of the Pennisetum species but also their potential for adaptation to different environmental conditions. The chloroplast genome, being central to the plant’s photosynthetic machinery, plays a crucial role in how these plants respond to climatic changes, water availability, and soil conditions (Raveendar et al., 2019; Razi and Muneer, 2021).

Furthermore, this study provides valuable genomic resources that directly support plant breeding and conservation efforts in Pennisetum (Xu et al., 2021). For plant breeding, the applications are multifaceted. First, the dense set of SSR and SNP markers identified in the chloroplast genomes can serve as powerful tools for tracing cytoplasmic lineage (Provan et al., 2001). This is particularly crucial for developing and maintaining cytoplasmic male sterility (CMS) systems in crops like pearl millet (*P. glaucum*), a technology essential for efficient hybrid seed production (Wang et al., 2024; Li B. et al., 2020). Second, a comprehensive understanding of the chloroplast genetic diversity across the genus allows breeders to strategically select wild relatives for introgression programs, aiming to transfer beneficial traits such as drought tolerance or disease resistance from the chloroplasts of wild species into elite cultivars (Xu et al., 2021). Finally, the identified DNA barcodes can be used for rapid and accurate germplasm authentication, ensuring the genetic integrity of breeding stocks and preventing misidentification (Tang et al., 2022). For conservation, these markers are invaluable for assessing the genetic diversity of wild populations and designing effective strategies to preserve the rich Pennisetum gene pool.

In conclusion, the comparative genomic analysis of Pennisetum chloroplast genomes offers a comprehensive view of their evolutionary and functional landscape. It highlights the balance between conservation and variation, underscoring the importance of chloroplast genome studies in understanding plant evolution, diversity, and the mechanisms underlying their adaptation and survival in varying ecosystems.

## Data Availability

The datasets presented in this study can be found in online repositories. The chloroplast genome sequences are available at NCBI (https://www.ncbi.nlm.nih.gov/nuccore/) under the project numbers: PP159189, PP159190, PP159191, PP159192, PP159193, PP159194, PP159195, PP159196, PP159197, PP159198, and PP159199.

## References

[B1] Abdi S. Dwivedi A. Kumar S. Bhat V. (2019). Development of EST-SSR markers in Cenchrus ciliaris and their applicability in studying the genetic diversity and cross-species transferability. J. Genet. 98, 101–116. 10.1007/s12041-019-1142-x 31767818

[B2] Bankevich A. Nurk S. Antipov D. Gurevich A. A. Dvorkin M. Kulikov A. S. (2012). SPAdes: a new genome assembly algorithm and its applications to single-cell sequencing. J. Comput. Biol. 19, 455–477. 10.1089/cmb.2012.0021 22506599 PMC3342519

[B3] Bolger A. M. Lohse M. Usadel B. (2014). Trimmomatic: a flexible trimmer for illumina sequence data. Bioinformatics 30, 2114–2120. 10.1093/bioinformatics/btu170 24695404 PMC4103590

[B4] Borsch T. Quandt D. (2009). Mutational dynamics and phylogenetic utility of noncoding chloroplast DNA. Plant Syst. Evol. 282, 169–199. 10.1007/s00606-009-0210-8

[B5] Brunken J. N. (1977). A systematic study of Pennisetum sect. Pennisetum (Gramineae). Am. J. Bot. 64, 161–176. 10.1002/j.1537-2197.1977.tb15715.x

[B6] Burton G. W. Powell J. B. (1968). Pearl millet breeding and cytogenetics. Adv. Agron. 20, 49–89. 10.1016/s0065-2113(08)60854-8

[B7] Cahoon A. B. Sharpe R. M. Mysayphonh C. Thompson E. J. Ward A. D. Lin A. (2010). The complete chloroplast genome of tall fescue (lolium arundinaceum; poaceae) and comparison of whole plastomes from the family poaceae. Am. J. Bot. 97, 49–58. 10.3732/ajb.0900008 21622366

[B8] Daniell H. Lin C.-S. Yu M. Chang W.-J. (2016). Chloroplast genomes: diversity, evolution, and applications in genetic engineering. Genome Biol. 17, 134. 10.1186/s13059-016-1004-2 27339192 PMC4918201

[B9] De Las Rivas J. Lozano J. J. Ortiz A. R. (2002). Comparative analysis of chloroplast genomes: functional annotation, genome-based phylogeny, and deduced evolutionary patterns. Genome Res. 12, 567–583. 10.1101/gr.209402 11932241 PMC187516

[B10] Dong W. Liu J. Yu J. Wang L. Zhou S. (2012). Highly variable chloroplast markers for evaluating plant phylogeny at low taxonomic levels and for DNA barcoding. PloS one 7, e35071. 10.1371/journal.pone.0035071 22511980 PMC3325284

[B11] Doyle J. J. Davis J. I. Soreng R. J. Garvin D. Anderson M. J. (1992). Chloroplast DNA inversions and the origin of the grass family (poaceae). Proc. Natl. Acad. Sci. 89, 7722–7726. 10.1073/pnas.89.16.7722 1502190 PMC49783

[B12] Frazer K. A. Pachter L. Poliakov A. Rubin E. M. Dubchak I. (2004). VISTA: computational tools for comparative genomics. Nucleic acids Res. 32, W273–W279. 10.1093/nar/gkh458 15215394 PMC441596

[B13] Godden G. T. Jordon-Thaden I. E. Chamala S. Crowl A. A. García N. Germain-Aubrey C. C. (2012). Making next-generation sequencing work for you: approaches and practical considerations for marker development and phylogenetics. Plant Ecol. Divers. 5, 427–450. 10.1080/17550874.2012.745909

[B14] Greiner S. Lehwark P. Bock R. (2019). OrganellarGenomeDRAW (OGDRAW) version 1.3. 1: expanded toolkit for the graphical visualization of organellar genomes. Nucleic acids Res. 47, W59–W64. 10.1093/nar/gkz238 30949694 PMC6602502

[B15] Gupta M. K. Donde R. Sabarinathan S. Gouda G. Dash G. K. Pati P. (2021). Microsatellite markers from whole genome and transcriptomic sequences. Bioinforma. Rice Res. Theor. Tech., 387–412. 10.1007/978-981-16-3993-7_18

[B16] Haq S. U. Dhingra P. Sharma M. Kothari S. Kachhwaha S. (2021). Plasticity of tandem repeats in expressed sequence tags of angiospermic and non-angiospermic species: insight into cladistic, phenetic, and elementary explorations. J. Appl. Biol. Biotechnol. 9, 36–59. 10.7324/JABB.2021.9204

[B17] Howe C. J. Barbrook A. C. Koumandou V. L. Nisbet R. E. R. Symington H. A. Wightman T. F. (2003). Evolution of the chloroplast genome. Philosophical Trans. R. Soc. Lond. Ser. B Biol. Sci. 358, 99–107. 10.1098/rstb.2002.1176 12594920 PMC1693101

[B18] Hu S. Sablok G. Wang B. Qu D. Barbaro E. Viola R. (2015). Plastome organization and evolution of chloroplast genes in cardamine species adapted to contrasting habitats. BMC genomics 16, 306–314. 10.1186/s12864-015-1498-0 25887666 PMC4446112

[B19] Hu C. Jiao Z. Deng X. Tu X. Lu A. Xie C. (2022). The ecological adaptation of the unparalleled plastome character evolution in slipper orchids. Front. Plant Sci. 13, 1075098. 10.3389/fpls.2022.1075098 36605947 PMC9808092

[B20] Huang D. I. Cronk Q. C. (2015). Plann: a command‐line application for annotating plastome sequences. Appl. plant Sci. 3, 1500026. 10.3732/apps.1500026 26312193 PMC4542940

[B21] Huang Y.-Y. Cho S.-T. Haryono M. Kuo C.-H. (2017). Complete chloroplast genome sequence of common bermudagrass (Cynodon dactylon (L.) Pers.) and comparative analysis within the family Poaceae. PloS one 12, e0179055. 10.1371/journal.pone.0179055 28617867 PMC5472289

[B22] Kumar S. Saxena S. Rai A. Radhakrishna A. Kaushal P. (2019). Ecological, genetic, and reproductive features of cenchrus species indicate evolutionary superiority of apomixis under environmental stresses. Ecol. Indic. 105, 126–136. 10.1016/j.ecolind.2019.05.036

[B23] Kurtz S. Choudhuri J. V. Ohlebusch E. Schleiermacher C. Stoye J. Giegerich R. (2001). REPuter: the manifold applications of repeat analysis on a genomic scale. Nucleic acids Res. 29, 4633–4642. 10.1093/nar/29.22.4633 11713313 PMC92531

[B24] Li X. Yang Y. Henry R. J. Rossetto M. Wang Y. Chen S. (2015). Plant DNA barcoding: from gene to genome. Biol. Rev. 90, 157–166. 10.1111/brv.12104 24666563

[B25] Li D.-M. Zhu G.-F. Xu Y.-C. Ye Y.-J. Liu J.-M. (2020). Complete chloroplast genomes of three medicinal alpinia species: genome organization, comparative analyses and phylogenetic relationships in family zingiberaceae. Plants 9, 286. 10.3390/plants9020286 32102387 PMC7076362

[B26] Li C. C. Zheng Y. Huang P. (2020). Molecular markers from the chloroplast genome of rose provide a complementary tool for variety discrimination and profiling. Sci. Rep. 10, 12188. 10.1038/s41598-020-68092-1 32699274 PMC7376030

[B27] Li B. B. Lin F. Huang P. Guo W. Zheng Y. (2020). Development of nuclear SSR and chloroplast genome markers in diverse Liriodendron chinense germplasm based on low-coverage whole genome sequencing. Biol. Res. 53, 21. 10.1186/s40659-020-00289-0 32410692 PMC7227249

[B28] Mader M. Pakull B. Blanc-Jolivet C. Paulini-Drewes M. Bouda Z.H.-N. Degen B. (2018). Complete chloroplast genome sequences of four meliaceae species and comparative analyses. Int. J. Mol. Sci. 19, 701. 10.3390/ijms19030701 29494509 PMC5877562

[B29] Martel E. De Nay D. Siljak-Yakoviev S. Brown S. Sarr A. (1997). Genome size variation and basic chromosome number in pearl millet and fourteen related pennisetum species. J. Hered. 88, 139–143. 10.1093/oxfordjournals.jhered.a023072

[B30] McInerney J. O. (1998). GCUA: general codon usage analysis. Bioinforma. Oxf. Engl. 14, 372–373. 10.1093/bioinformatics/14.4.372 9632833

[B31] Meng X.-X. Xian Y.-F. Xiang L. Zhang D. Shi Y.-H. Wu M.-L. (2018). Complete chloroplast genomes from sanguisorba: identity and variation among four species. Molecules 23, 2137. 10.3390/molecules23092137 30149578 PMC6225366

[B32] Ohyama K. Fukuzawa H. Kohchi T. Shirai H. Sano T. Sano S. (1986). Chloroplast gene organization deduced from complete sequence of liverwort Marchantia polymorpha chloroplast DNA. Nature 322, 572–574. 10.1038/322572a0

[B33] Presting G. G. (2006). Identification of conserved regions in the plastid genome: implications for DNA barcoding and biological function. Botany 84, 1434–1443. 10.1139/b06-117

[B34] Provan J. Powell W. Hollingsworth P. M. (2001). Chloroplast microsatellites: new tools for studies in plant ecology and evolution. Trends Ecol. Evol. 16, 142–147. 10.1016/s0169-5347(00)02097-8 11179578

[B35] Raveendar S. Lee G. Lee K. Shin M. Lee J. Lee S. (2019). The complete chloroplast genome of pearl millet (Pennisetum glaucum (L.) R. Br.) and comparative analysis within the family Poaceae. Cereal Res. Commun. 47, 1–10. 10.1556/0806.46.2018.064

[B36] Razi K. Muneer S. (2021). Drought stress-induced physiological mechanisms, signaling pathways and molecular response of chloroplasts in common vegetable crops. Crit. Rev. Biotechnol. 41, 669–691. 10.1080/07388551.2021.1874280 33525946

[B37] Sathyamurthy D. Mannu J. Natesan S. Nathan B. Nallusamy S. Narayanan M. B. (2024). Comparative chloroplast genome analysis of six millet species along with related Poaceae family members. Nucl. 68, 13–24. 10.1007/s13237-023-00464-0

[B38] Sharma S. Sharma R. Pujar M. Yadav D. Yadav Y. Rathore A. (2021). Use of wild Pennisetum species for improving biotic and abiotic stress tolerance in pearl millet. Crop Sci. 61, 289–304. 10.1002/csc2.20408

[B39] Sharp P. M. Li W.-H. (1987). The codon adaptation index-a measure of directional synonymous codon usage bias, and its potential applications. Nucleic acids Res. 15, 1281–1295. 10.1093/nar/15.3.1281 3547335 PMC340524

[B40] Shinozaki K. Ohme M. Tanaka M. Wakasugi T. Hayashida N. Matsubayashi T. (1986). The complete nucleotide sequence of the tobacco chloroplast genome: its gene organization and expression. EMBO J. 5, 2043–2049. 10.1002/j.1460-2075.1986.tb04464.x 16453699 PMC1167080

[B41] Shivhare R. Lata C. (2017). Exploration of genetic and genomic resources for abiotic and biotic stress tolerance in pearl millet. Front. plant Sci. 7, 2069. 10.3389/fpls.2016.02069 28167949 PMC5253385

[B42] Tang D. Lin Y. Wei F. Quan C. Wei K. Wei Y. (2022). Characteristics and comparative analysis of mesona chinensis Benth chloroplast genome reveals DNA barcode regions for species identification. Funct. Integr. Genomics 22, 467–479. 10.1007/s10142-022-00846-8 35318559

[B43] Tonti Filippini J. Nevill P. G. Dixon K. Small I. (2017). What can we do with 1000 plastid genomes? Plant J. 90, 808–818. 10.1111/tpj.13491 28112435

[B44] Wang X. Wang L. (2016). GMATA: an integrated software package for genome-scale SSR mining, marker development and viewing. Front. plant Sci. 7, 1350. 10.3389/fpls.2016.01350 27679641 PMC5020087

[B45] Wang Q. Niu Z. Li J. Zhu K. Chen X. (2020). The complete chloroplast genome sequence of the Chinese endemic species Sorbus setschwanensis (Rosaceae) and its phylogenetic analysis. Nordic J. Bot. 38, njb.02532. 10.1111/njb.02532

[B46] Wang Z. Cai Q. Wang Y. Li M. Wang C. Wang Z. (2022). Comparative analysis of codon bias in the chloroplast genomes of theaceae species. Front. Genet. 13, 824610. 10.3389/fgene.2022.824610 35360853 PMC8961065

[B47] Wang R. Yang Y. Tian H. Yi H. Xu L. Lv Y. (2024). A scalable and robust chloroplast genotyping solution: development and application of SNP and InDel markers in the maize chloroplast genome. Genes 15, 293. 10.3390/genes15030293 38540352 PMC10970264

[B48] Xiao-Ming Z. Junrui W. Li F. Sha L. Hongbo P. Lan Q. (2017). Inferring the evolutionary mechanism of the chloroplast genome size by comparing whole-chloroplast genome sequences in seed plants. Sci. Rep. 7, 1555. 10.1038/s41598-017-01518-5 28484234 PMC5431534

[B49] Xu J. Liu C. Song Y. Li M. (2021). Comparative analysis of the chloroplast genome for four Pennisetum species: molecular structure and phylogenetic relationships. Front. Genet. 12, 687844. 10.3389/fgene.2021.687844 34386040 PMC8354216

[B50] Yadav O. Rai K. Gupta S. (2012). “Pearl millet: genetic improvement in tolerance to abiotic stresses,” in Improving crop productivity in sustainable agriculture, 261–288.

[B51] Yadav O. Gupta S. Rai K. (2024). “Milestones in biology, genetics, and breeding of Pearl Millet,” in Pearl millet in the 21st century: food-Nutrition-Climate resilience-improved livelihoods (Springer), 61–86.

[B52] Zhang Y. Nie X. Jia X. Zhao C. Biradar S. S. Wang L. (2012). Analysis of codon usage patterns of the chloroplast genomes in the Poaceae family. Aust. J. Bot. 60, 461–470. 10.1071/bt12073

[B53] Zhang D. Li K. Gao J. Liu Y. Gao L.-Z. (2016). The complete plastid genome sequence of the wild rice Zizania latifolia and comparative chloroplast genomics of the rice tribe Oryzeae, Poaceae. Front. Ecol. Evol. 4, 88. 10.3389/fevo.2016.00088

[B54] Zhou M. Long W. Li X. (2008). Patterns of synonymous codon usage bias in chloroplast genomes of seed plants. For. Stud. China 10, 235–242. 10.1007/s11632-008-0047-1

[B55] Zurawski G. Clegg M. T. (1987). Evolution of higher-plant chloroplast DNA-encoded genes: implications for structure-function and phylogenetic studies. Annu. Rev. plant physiology 38, 391–418. 10.1146/annurev.pp.38.060187.002135

